# Prevalence of autoimmune diabetes among Aboriginal Australians: the Fremantle Diabetes Study Phase II

**DOI:** 10.1111/imj.70029

**Published:** 2025-03-18

**Authors:** Timothy M. E. Davis, Christine S. Bundell, Stephen A. Paul Chubb, Daniel McAullay, Wendy A. Davis

**Affiliations:** ^1^ Medical School University of Western Australia, Fremantle Hospital Fremantle Western Australia Australia; ^2^ Department of Endocrinology and Diabetes Fiona Stanley and Fremantle Hospitals Group Perth Western Australia Australia; ^3^ Department of Clinical Immunology, PathWest Laboratory Medicine QEII Medical Centre Perth Western Australia Australia; ^4^ Department of Biochemistry, PathWest Laboratory Medicine Fiona Stanley Hospital Perth Western Australia Australia; ^5^ Kurongkurl Katitjin, Centre for Indigenous Australian Education and Research Edith Cowan University Perth Western Australia Australia

**Keywords:** autoimmune diabetes, indigenous, prevalence, type 1 diabetes, latent autoimmune diabetes of adults

## Abstract

The prevalence of autoimmune diabetes was assessed in 113 indigenous and 1555 non‐indigenous participants in the Fremantle Diabetes Study Phase II. Both type 1 diabetes (3.5% vs. 8.2%) and latent autoimmune diabetes of adults diagnosed based on glutamic acid decarboxylase antibody (GADA) positivity (1.0% vs. 5.7%) were lower in Aboriginal participants (*P* = 0.101 and 0.039 respectively). Six Aboriginals with GADA‐negative type 2 diabetes were positive for tyrosine phosphatase‐related islet antigen 2 antibodies but did not exhibit relative insulin deficiency.

There are few and conflicting data relating to the frequency of autoimmune diabetes in Australian Aboriginal and Torres Strait Islander communities. A review published in 2016 concluded that type 1 diabetes was relatively uncommon in the Aboriginal and Torres Strait Islander population based on Australian Institute of Health and Welfare data from 2010.^1^ Administrative data collected in the Northern Territory from 2012 to 2019 suggested that 1.0% of Aboriginal people with diabetes were classified as type 1^2^ compared with a much higher overall national estimate for 2011–2012 of 7.9%.^3^ The percentages of type 1 diabetes represented by Aboriginal and Torres Strait Islander children and young adults in the Australasian Diabetes Data Network up to 2020^4^ and in children diagnosed with type 1 diabetes in Western Australia between 1999 and 2019^5^ (1.9% and 2.0% respectively) were less than half the percentage of the general population aged <20 years identifying as Aboriginal and Torres Strait Islander in the 2021 national census (≈5%).^6^ However, a study of reported cases of type 1 diabetes in New South Wales in 1990–1991 found no significant difference in incidence between children from Aboriginal and Torres Strait Islander and other ethnic/racial groups.^7^

There are several limitations associated with the available data. Potential misclassification of diabetes type due to a lack of objective criteria, including islet autoantibody titres and coding errors, is an acknowledged issue with administrative database studies,^2^, ^4^ including those based on National Diabetes Services Scheme data.^1^ More rigorous studies employing the measurement of islet autoantibodies are restricted to children or young adults,^5^, ^7^ even though a significant proportion of cases are diagnosed as type 1 diabetes in adulthood, including latent autoimmune diabetes of adults (LADA). LADA is associated with glutamic acid decarboxylase (GAD) antibody positivity and considered a slowly evolving form of type 1 diabetes.^8^ The aim of the present study was, therefore, to determine the relative prevalence of type 1 diabetes and LADA in Aboriginal participants in the community‐based Fremantle Diabetes Study Phase II (FDS2).

The FDS2 is a longitudinal observational cohort study involving representative participants recruited from a sociodemographically representative urban Australian setting between 2008 and 2011.^9^, ^10^ The study design and conduct involved an Aboriginal researcher (DM), and two Aboriginal health workers were employed to facilitate recruitment of, and data collection from, Aboriginal participants. The basic demographic and diabetes‐specific characteristics of individuals recruited to FDS2 were similar to those of eligible people from the catchment area who were not recruited.^9^ All FDS2 participants were invited to face‐to‐face assessments at entry and then biennially over the next 6 years. At each assessment, a standardised comprehensive questionnaire was completed and a physical examination was performed, and blood and urine samples were sent for fasting biochemical tests performed in a single nationally accredited laboratory (PathWest, Fremantle and Nedlands Western Australia).

Racial/ethnic background was categorised according to self‐selection, country/countries of birth and parents'/grandparents' birth and language(s) spoken at home as Anglo‐Celt, Southern European, Other European, Asian, Aboriginal or mixed/other.^9^ In line with Australian legal rulings and a range of other studies of Aboriginal and Torres Strait Islander Australians with diabetes,^11^, ^12^, ^13^ self‐identification and acceptance by the local community were used as the primary criteria for Aboriginality.^14^ No FDS2 participant self‐identified as a Torres Strait Islander. The FDS2 was approved by the South Metropolitan Area Health Service Human Research Ethics Committee (reference 07/397) and all participants gave written informed consent.

Type of diabetes was ascertained from study investigator review of baseline data, including participant self‐report, documentation by health professionals, treatment history, anthropometric measures including body mass index (BMI) and the results of any relevant prior laboratory tests. For those with clinically defined type 2 diabetes, participants at high risk for monogenic diabetes were genotyped for relevant mutations^15^, ^16^ and baseline sera were initially assayed for GAD autoantibodies (GADA) by radioimmunoassay (RIA).^17^ A second‐generation enzyme‐linked immunosorbent assay (ELISA; GAD65, RSR Limited, Cardiff, UK) was used subsequently to recheck all first‐generation GAD antibody‐positive and a random sample of antibody‐negative results. The baseline samples for these follow‐up tests had been kept at −80°C until they were assayed. GAD65 positivity was defined as ≥5 U/mL with mean inter‐ and intra‐assay coefficients of variation of 6.5% and 6.4% respectively. The RIA‐GADA has been shown to detect both disease‐irrelevant and disease‐relevant signals, but the ELISA‐GAD assay detects disease‐relevant signals exclusively.^18^ Tyrosine phosphatase‐related islet antigen 2 (IA‐2) antibodies were measured by RIA and zinc transporter Zn8 antibodies by commercially available ELISA (both RSR Limited, Cardiff, UK) in the Aboriginal participants. The threshold for the RIA IA‐2 assay was >1.0 U/mL, which is equivalent to >7.5 U/mL for the more recent ELISA IA‐2 assays, and the assay upper limit was 50 U/mL.

There were 113 Aboriginal FDS2 participants (mean age 53.7 years, 36.3% males). Of these four (3.5%) had been diagnosed clinically with type 1 diabetes (insulin requiring from diagnosis), 108 (95.6%) with type 2 diabetes and 1 (0.9%) with type 3C diabetes (Table 1). Using the first‐generation GAD antibody assay and baseline sera from 103 Aboriginal participants with clinically diagnosed type 2 diabetes and an available sample, seven (6.8%) were GAD antibody positive and thus were considered to have LADA, but this reduced to one (1.0%) using the second‐generation ELISA assay (Table 1). There was a between‐assay concordance of 83.2% with most discordant results for low positive values by the RIA method. Of 1555 non‐Aboriginal FDS2 participants, 128 (8.2%) had type 1 diabetes (*P* = 0.101 vs. Aboriginal sample by Fisher's exact test); of those with available samples (*n* = 1554) and excluding type 1 diabetes, secondary diabetes, neonatal and monogenic diabetes (*n* = 152), 80 (5.7%) had LADA based on the second‐generation ELISA GAD antibody assay (*P* = 0.039 vs. Aboriginal sample; see Table 1). Six of the 103 Aboriginal participants with clinically diagnosed type 2 diabetes (5.8%) were positive for IA‐2 at generally low‐level titres, but all were negative for GADA. If these six individuals had LADA, the prevalence of LADA in those with clinically diagnosed type 2 diabetes increased to 6.8%. None of the Aboriginal participants was positive for ZnT8 antibodies.

**Table 1:** Summary of diabetes‐related autoimmunity among Aboriginal and non‐Aboriginal participants in Fremantle diabetes study phase II living with diabetes in the study area at baseline

	Aboriginal	Non‐Aboriginal	*P*‐value	Comment
Total	113 (6.7%)	1555 (93.3%)		
Type 1 diabetes	4/113 (3.5%)	128/1555 (8.2%)	0.101	
LADA (second generation assay)	1/103† (1.0%)	80/1402‡ (5.7%)	0.039	Excluding type 1/monogenic forms/secondary diabetes including type 3C
Total autoimmune diabetes	5/108† (4.6%)	208/1554‡ (13.4%)	0.007	Excluding IA‐2 seropositives
Total autoimmune diabetes	11/108† (10.2%)	—		Including IA‐2 seropositives

† GADA and IA‐2 unmeasured in five participants without type 1 diabetes.

‡ GADA unmeasured in one participant without type 1 diabetes.

A comparison of characteristics of the six Aboriginal FDS2 participants with a positive IA‐2 antibody titre and those who were IA‐2 seronegative is summarised in Table 2. The seropositive participants had longer diabetes duration, but other FDS2 baseline variables were not significantly different. In particular, only one IA‐2 seropositive participant had a positive type 1 diabetes genetic risk score,^19^ and none was insulin‐treated from diagnosis or at FDS2 baseline assessment, consistent with serum C‐peptide concentrations that were mostly >0.20 nmol/L (Table 2).

**Table 2:** Baseline characteristics of Aboriginal FDS2 participants who were IA‐2 seropositive compared with those who were IA‐2 seronegative

	IA‐2 seropositive	IA‐2 seronegative	*P*‐value
Number	6	96	
IA‐2 titre (U/mL)	1.23 (1.11–1.32)	0 (0–0)	<0.001
Age (years)	53.8 ± 3.2	54.6 ± 12.2	0.666
Age at diagnosis (years)	36.0 ± 9.3	44.4 ± 14.5	0.170
Diabetes duration (years)	17.4 (9.0–27.3)	7.3 (3.0–16.0)	0.049
BMI (kg/m^2^)	32.7 ± 9.9	31.8 ± 7.1	0.778
HbA_1c_ (%)	8.6 (6.8–10.8)	8.1 (6.6–10.6)	0.983
HbA_1c_ (mmol/mol)	70 (51–94)	65 (49–92)	0.983
Serum glucose (mmol/L)	9.2 (8.3–10.9)	7.9 (6.2–12.4)	0.408
Serum C‐peptide (nmol/L)	0.50 (0.26–0.98)	0.28 (0.08–0.95)	0.249
Type 1 diabetes genetic risk score >0.28 (n/N; %)	1/6 (16.7)	1/86 (1.2)	0.127
Insulin treatment from diagnosis (%)	0/5 (0)	2/72 (2.8)	>0.999
Insulin treatment at FDS2 entry (%)	0/6 (0)	23/95 (24.2)	0.332

## Discussion

Based on a clinical diagnosis of type 1 diabetes and the second‐generation ELISA GAD assay results, autoimmune diabetes was approximately half as prevalent in Aboriginal versus non‐Aboriginal adult participants from the representative community‐based FDS2. This is consistent with most,^1^, ^2^, ^4^, ^5^ but not all,^7^ previous studies from a variety of Australian geographical contexts and employing heterogeneous methods of ascertainment. The presence of isolated IA‐2 seropositivity among some Aboriginal participants raises the possibility that these individuals had an autoimmune component to their diabetes pathogenesis, but their clinical characteristics suggest that the contribution of IA‐2 seropositivity to pancreatic beta cell dysfunction was minimal.

There is a variable relationship between Aboriginal and Torres Strait Islander ethnicity and other autoimmune diseases. Aboriginal and Torres Strait Islander peoples have increased frequencies of rheumatic fever, systemic lupus erythematosus and post‐streptococcal glomerulonephritis, but lower rates of a range of other immune‐mediated conditions such as rheumatoid arthritis, multiple sclerosis, biliary cirrhosis, coeliac disease and pernicious anaemia.^20^, ^21^ This suggests that there is no consistent underlying race‐specific immunological mechanism, with each disease requiring relevant race/ethnicity epidemiological data. Indeed, the need for more robust studies in Aboriginal and Torres Strait Islander peoples has been highlighted,^20^, ^21^ especially for autoimmune thyroid disease for which there are currently no published data. The well‐curated community‐based FDS2 cohort provides informative data in the case of autoimmunity‐associated diabetes in adults.

Although we did not measure IA‐2 antibodies in our non‐Aboriginal participants, in a recent study comparing autoimmune diabetes in adults from Germany and Singapore,^22^ GAD antibodies were more frequent in the European cohort, but IA‐2 antibody positivity was highest in the three ethnic groups in Singapore (Indian, Malay and Chinese). The positive IA‐2 titres were, as in the present study, low level in the majority of participants. The authors did not speculate as to why there was this racial difference, other than suggesting that there are ethnic‐specific differences in antibody profiles.^22^ There is evidence that IA‐2 seropositivity on its own may be an important predictor of impending type 1 diabetes in siblings of patients,^23^ but our IA‐2 seropositive FDS2 Aboriginal participants were non‐insulin‐treated and did not show comparatively depressed serum C‐peptide concentrations despite a relatively long diabetes duration. Given that young Australian Aboriginal people with type 2 diabetes can present in diabetic ketoacidosis,^24^ isolated IA‐2 seropositivity in this situation may not indicate the need for continued insulin therapy beyond acute management. The present and other data^22^ could also reflect ethnicity‐specific differences in the threshold for IA‐2 positivity that would only be revealed by large scale studies including individuals without diabetes.

In conclusion, our data from representative urban community‐based individuals with well‐characterised diabetes show that autoimmune diabetes, whether type 1 or LADA, has a relatively reduced prevalence in Aboriginal people. However, the frequency and clinical consequences of isolated IA‐2 seropositivity in this group should be examined in other cohorts.
